# Amphipathic tail-anchoring peptide is a promising therapeutic agent for prostate cancer treatment

**DOI:** 10.18632/oncotarget.2301

**Published:** 2014-07-31

**Authors:** Gejing De, Jae-Kyun Ko, Tao Tan, Hua Zhu, Haichang Li, Jianjie Ma

**Affiliations:** ^1^ Department of Surgery, Davis Heart and Lung Research Institute, The Ohio State University Wexner Medical Center, Columbus, OH, USA; ^2^ Department of Physiology and Biophysics, Rutgers University-Robert Wood Johnson Medical School, Piscataway, NJ, USA; ^3^ Mutagenex Inc., 1 Jill Court, Hillsborough, NJ, USA; ^4^ TRIM-edicine, Inc, Columbus, OH, USA

**Keywords:** peptide, ATAP-iRGD, cancer therapy, integrin, apoptosis, mitochondria

## Abstract

Amphipathic tail-anchoring peptide (ATAP) derived from the human anti-apoptotic protein Bfl-1 is a potent inducer of apoptosis by targeting mitochondria permeability transition. By linking ATAP to an internalizing RGD peptide (iRGD), selective targeting for ATAP to tumor cell was achieved. Confocal fluorescence microscopy showed that ATAP-iRGD could penetrate into cancer cells and distribute along the mitochondria network. ATAP-iRGD triggered mitochondria-dependent cell death through release of cytochrome c. In an effort to promote ATAP-iRGD physiochemical properties to approach clinic application, amino acid substitution and chemical modification were made with ATAP-iRGD to improve its bioactivity. One of these modified peptides, ATAP-iRGD-M8, was with improved stability and aqueous solubility without compromising *in vitro* cytotoxicity in cultured cancer cells. *In vivo* xenograft studies with multiple prostate cancer cell lines showed that intravenous administration of ATAP-iRGD-M8 suppressed tumor growth. Toxicological studies revealed that repetitive intravenous administration of ATAP-iRGD-M8 did not produce significant toxicity in the SV129 mice. Our data suggest that ATAP-iRGD-M8 is a promising agent with high selectivity and limited systemic toxicity for prostate cancer treatment.

## INTRODUCTION

A major focus in current cancer research is to develop small molecules or peptides as potential therapeutic agents or diagnostic tools for cancer treatment [1-5]. The peptide drugs either serve as cytotoxic agents to directly suppress cancer cell growth or act as carriers for targeting of cytotoxic agents to tumor cells [3, 4, 6]. Compared with conventional chemotherapy or radiotherapy agents, peptide drugs have the advantage of tissue-specific targeting, thus off-target effects could be minimized [7-10]. Previous studies have shown that a tripeptide motif, RGD (Arg-Gly-Asp) or NGR (Asn-Gly-Arg), possessed strong affinity to interact with cell surface membrane integrin receptor to allow for homing of cytotoxic cargoes to tumor blood vessels [11-14]. RGD-mediated recognition of tumor cells could also be used as imaging tools for cancer diagnosis [15-17]. Recently, a disulfide-based cyclic RGD peptide referred to as internalizing RGD (iRGD) has shown improved efficacy in selectively delivering therapeutic or imaging agents to tumor site [17]. iRGD contained a sequence of nine amino acids, CRGDKGPDC, that facilitates interaction with neuropilin-1 receptor on the target tumor cells and thereby increase cell membrane permeability [18, 19]. Coating of nanoparticles containing paclitaxel with iRGD could enhance homing of the chemotherapeutic agent to tumor tissues [18], and co-administration of iRGD with doxorubicin and trastuzumab improved efficacy of these cancer drugs [20].

Mitochondrion is an essential intracellular organelle participating in regulation of cell growth and death, and has been a prime target for cytotoxic agents in cancer treatment [21-23]. For example, (KLAKLAK)_2_ is an antimicrobial peptide that target negatively charged mitochondria membrane [24, 25]. This peptide has low mammalian cell toxicity because it does not interfere with the zwitterionic plasma membrane of eukaryotic cells [24]. When internalized inside the cell, the D-(KLAKLAK)_2_ peptide causes disruption of the mitochondrial membrane potential and induces cell death. A bi-functional peptide RGD4C-D-(KLAKLAK)_2_, with the RGD motif added to the amino-terminus can selectively target tumorigenic endothelial cells that express high levels of integrinαVβ3 receptors [25, 26]. As another example, several studies have shown that peptides derived from the BH3 domain of pro-apoptotic Bcl-2 family members induce apoptosis of cancer cells by either directly binding and activating Bax and/or Bak or competing with anti-apoptotic Bcl-2 proteins and consequently sensitizes cells to apoptosis [27-29]. However, the efficacy of currently available BH3 peptides or chemical mimetics are limited by the high levels of Bcl-2 family proteins that are commonly found in cancer cells [30-32].

Previously, our group identified a novel mitochondrial targeting peptide, an amphipathic tail-anchoring peptide (ATAP), which triggers potent mitochondria-dependent apoptosis [33]. ATAP selectively target to mitochondria and induce cytochrome c release through perturbation of mitochondria membrane permeability [33-35]. Different from the BH3 peptide, the cytotoxic effects of ATAP do not require the presence of pro-apoptotic proteins such as Bak or Bax, and is not influenced by the content of anti-apoptotic proteins such as Bcl-2 or Bcl-xL [35]. These properties suggest that ATAP is unique among other cytotoxic mitochondrial pore forming peptides [29, 33, 35]. Based on these properties, we hypothesize that ATAP has the potential to be developed into a therapeutic agent to induce tumor cell death for cancer treatment.

While ATAP has potent pro-apoptotic activity, a major challenge is to ensure its recognition and penetration into tumor cells. Thus, ATAP peptides must be coupled to tumor targeting moiety that allows for receptor-mediated penetration into tumor cells. In this study, we conjugated ATAP with iRGD and tested its antitumor efficacy on prostate cancers by using both *in vitro* cell culture and *in vivo* xenograft mouse model. In order to improve stability and solubility of ATAP-iRGD, we generated a series of substitutive mutations. We found one of these mutations, ATAP-iRGD-M8 exhibited better stability and solubility as compared with ATAP-iRGD, while still maintains similar cytotoxicity in cancer cells. Our data showed that ATAP-iRGD is highly efficient in suppression of prostate tumor growth with limited systemic toxicity and ATAP-iRGD-M8 is a potentially better candidate for cancer treatment.

## RESULTS

### Tumor-targeting anticancer peptide design

**Figure 1:**
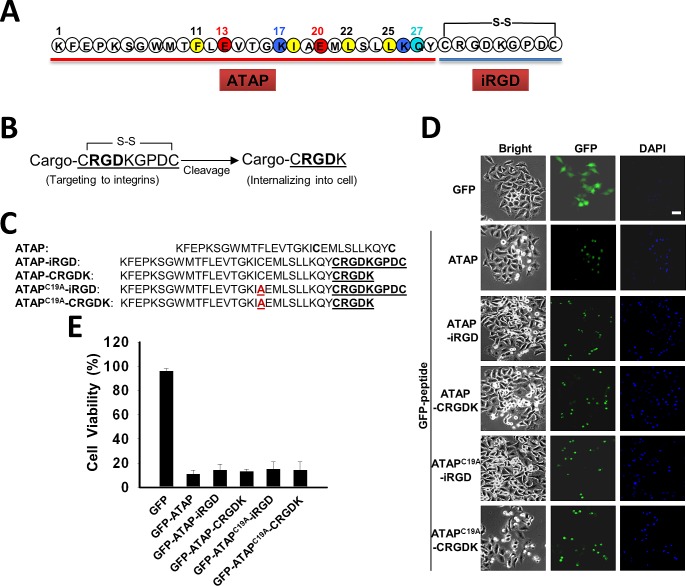
ATAP-iRGD fusion peptides maintain pro-apoptotic function (A) ATAP is coupled with the disulfide-based cyclic RGD moiety (iRGD) to form a chimeric peptide ATAP-iRGD. (B) Schematic representation of iRGD to illustrate exposure of the [CRGDK] element after proteolytic cleavage. (C) ATAP-iRGD peptide, related mutations ATAP^C19A^-iRGD, and the cleavage products ATAP-CRGDK, ATAP^C19A^-CRGDK are compared with the wild-type sequence (mutation is highlighted by red color). (D) HeLa cells were transfected with plasmids expressing GFP-ATAP and GFP-ATAP related plasmids. At 24 hours after transfection, cells were treated with DAPI and observed under fluorescence microscope. Representative photographs showing GFP- or DAPI-positive cells in the same field. The scale bar represents 20 μm. (E) Percentage of surviving cells was determined by the ratio of GFP-positive cells without DAPI staining to total GFP-positive cells. About 200 cells from three different fields were scored. Data are expressed as the means ± SEM.

Our previous study showed that ATAP derived from Bfl-1 targets mitochondria to induce caspase-dependent apoptosis [33-35]. ATAP peptide is composed of an amphipathic α-helix (amino acids 7–27) flanked by an amino- and a carboxyl-terminal motif that contain the mitochondria-targeting moiety [33]. For homing of ATAP peptide to cancer cells, we conjugated the iRGD sequence, [CRGDKGPDC], to the carboxyl-terminal end of ATAP (Fig. 1A), based on the work of Sugahara et al [18].

The rationale behind the design for ATAP-iRGD is illustrated in Fig. 1B and 1C. Basically, we needed to ensure that fusion of ATAP with the iRGD sequence did not alter the pro-apoptotic function for ATAP. It is known that the iRGD sequence undergoes proteolysis at the cell surface to expose the cleavage product CRGDK (Fig. 1B), thereby facilitating interaction with neurophilin-1 for internalization of the ATAP peptide into the cell [19]. To test whether the final cleavage product, ATAP-CRGDK, can enter the cells and maintain potent pro-apoptotic activity, GFP-ATAP-iRGD, GFP-ATAP-CRGDK fusion constructs were generated and transfected into HeLa cells to determine its pro-apoptotic function, following our published protocol [33-35]. In addition, ATAP contains one Cys residue (at position 19), which may complicate the cyclic disulfide-bond formation between the two Cys in the iRGD sequence. Thus, we have substituted this Cys with Ala to generate the ATAP^C19A^-iRGD peptide. The various ATAP and iRGD combinations are shown in Fig. 1C. As shown in Fig. 1D, expression of GFP-ATAP-iRGD, GFP-ATAP^C19A^-iRGD, GFP-ATAP-CRGDK or GFP-ATAP^C19A^-CRGDK in HeLa cells all resulted in drastic reduction in cell viability, to a degree that is similar to that induced by GFP-ATAP (Fig. 1E). Thus, neither fusion with iRGD nor C19A mutation appeared to affect the pro-apoptotic function for ATAP. Based on these studies, we synthesized the ATAP-iRGD peptide with the following amino acid sequence,

KFEPKSGWMTFLEVTGKIAEMLSLLKQY CRGDKGPDC, where the intra-molecular disulfide bridge was formed between the two Cys residues at position 29 and 37 (Fig. 1A).

### Pro-apoptotic effects of ATAP-iRGD in different cancer cells

**Figure 2:**
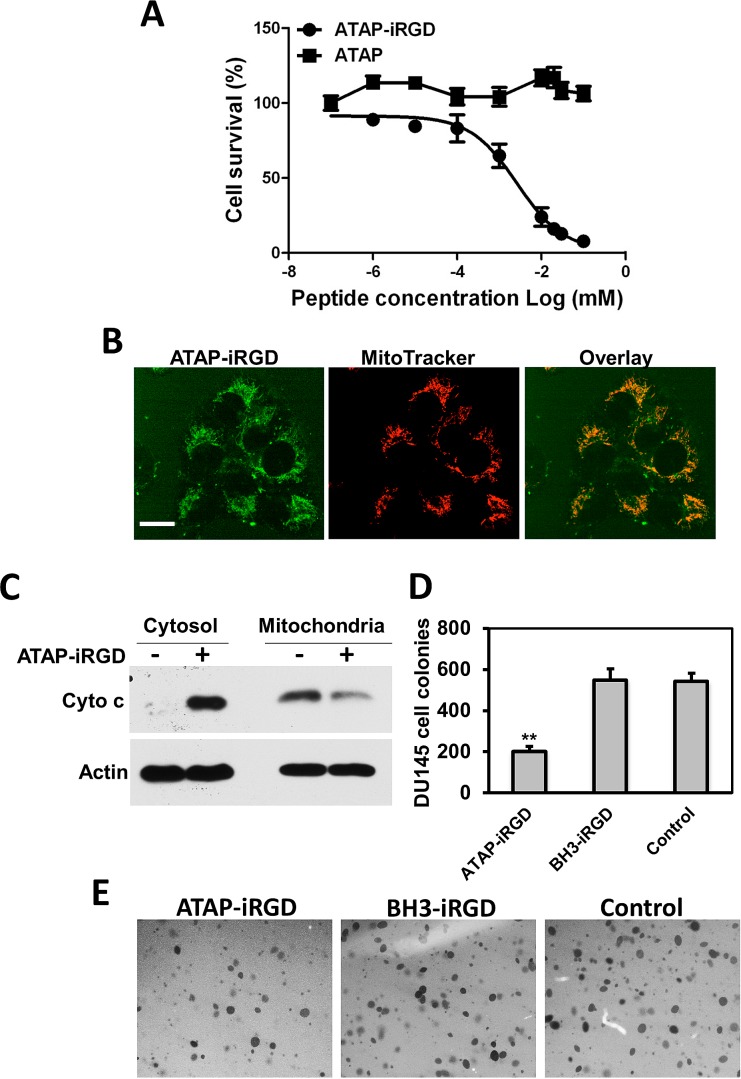
ATAP-iRGD targets mitochondria to cause apoptotic effects on tumor cells (A) MTT assay for ATAP and ATAP-iRGD in DU145 cells. (B) When applied externally to DU145 cells, the Dylight488-labeled ATAP-iRGD (*Green, left*) can enter into cells and associate with mitochondria labeled with Mito Tracker Red 633 (*Red*, *center*). Overlapping patterns for ATAP-iRGD and Mito Tracker was observed (*right*). These experiments were performed in the presence of 50 μM Q-VD-OPh to allow for observation of co-localization before induction of cell death. The scale bar represents 20 μm. (C) Western blots showing effect of ATAP-iRGD on cytochrome c release in DU145 cells. Incubation with ATAP-iRGD induces release of cytochrome c from mitochondria into the cytosol. (D) ATAP-iRGD inhibits DU145 cells colony formation, whereas no significant difference was observed between cells treated with control and BH3-iRGD peptide. Results were means of triplicates ± SEM from three independent experiments. (E) Representative pictures of colony formation assay with different treatments.

The efficacy for the synthetic ATAP-iRGD peptide to induce cancer cell death was evaluated in several cancer cell lines, including various prostate cancer cells (DU145, LNCaP and PC-3), U87 glioblastoma cells, MDA-MB-231 breast adenocarcinoma cells, and KYSE-150 esophageal squamous carcinoma cells. Based on MTT assay, ATAP-iRGD was effective in inducing cell death in these cell lines and the IC_50_ for ATAP-iRGD in these cells were summarized in Table 1. As shown in Fig. 2A, when DU145 cells were incubated with the unconjugated ATAP peptide (for 48 hours), the cells maintained viability over a wide concentration range of ATAP (0.01 – 100 μM). In contrast, incubation of DU154 cells with ATAP-iRGD led to dose-dependent cell death with an IC_50_ of 1.6±0.5 μM. This suggests that conjugation with iRGD facilitated delivery of ATAP into the tumor cells. Previous study by other investigators show that DU145 cells are resistant to apoptosis by BH3-derived peptide due to a deficiency in Bax expression [36]. Here we show that ATAP-iRGD had similar cytotoxicity in DU145 cells as wells as in KYSE-150, PC-3 and LNCaP cells. This result is consistent with our previous observation that the pro-apoptotic effect of ATAP-iRGD is Bax independent[35].

**Table 1:** Cytotoxic effect of ATAP-iRGD in different cancer cell lines

Cell me	Origin of tumor	IC_50_ (μM)
DU145	Prostate carcinoma	1.6±0.5
LNCap	Prostate carcinoma	7.1±1.7
PC-3	Prostate adenocarcinoma	9.2±1.6
U87	Glioblastoma	10.3±2.6
KYSE-150	Esophageal squamous carcinoma	4.4±0.5
K562	Chronic myelogenous leukemia	107±41
MDA-MB-231	Breast adenocarcinoma	5.9±1.1

Homing of iRGD to tumors cell is mediated by high expression of integrin αVβ3 on the cell surface membrane [18]. For testing the correlation between the level of integrin αVβ3 receptors and the efficacy for ATAP-iRGD to induce cell death, we chose a human chronic myelogenous leukemia cell line K562, which has been shown to express low level of integrin αVβ3[37]. As shown in Fig. S1, flow cytometry analysis confirmed that the low level of integrin αVβ3 was expressed in K562 cells. And MTT assay showed that K562 was more resistant to ATAP-iRGD treatment as compared to other cell lines (see Table 1). These results support a role for integrin receptor as a mediator to facilitate uptake of ATAP-iRGD into cancer cells.

For direct visualizing the uptake of ATAP-iRGD by tumor cells, Dylight488-labeled ATAP-iRGD peptide was applied to the culture medium surrounding DU145 cells. Confocal fluorescence microscopy imaging showed that ATAP-iRGD could effectively penetrate into cells and distribute along the mitochondria network. As shown in Fig. 2B, the green fluorescence of Dylight488-ATAP-iRGD showed overlapping patterns with mitochondrion-specific dye Mito Tracker Red, confirming that ATAP-iRGD displayed high selectivity in targeting to mitochondria. We used biochemical assay to determine if ATAP-iRGD can trigger mitochondria-dependent apoptosis. As shown in Fig. 2C, treatment of DU145 cells with 10 μM ATAP-iRGD caused significant amount of cytochrome c released from mitochondria into the cell cytosol, whereas release of cytochrome c was undetectable in untreated cell samples. This result indicates that ATAP-iRGD applied outside of the cell was able to enter the cells and target mitochondria to induce apoptosis.

For further quantification of the cytotoxic effect of ATAP-iRGD in DU145 cells, soft agar based cancer cell colony formation was used. For comparative purpose, DU145 cells were treated with either ATAP-iRGD or BH3-iRGD peptides. As shown in Fig. 2D, many visible colonies were developed from cancer cells grown in soft agar medium supplemented with BH3-iRGD peptide or normal medium, but fewer colonies were developed from cells cultured with ATAP-iRGD. These results show that ATAP-iRGD peptide is able to reduce the anchorage-independent cell growth in DU145 cells, which has previously been shown to be Bax deficient and resistant to treatment with BH3 mimetic ABT-737 [36].

### Improvement of peptide bioactivity and stability

**Figure 3:**
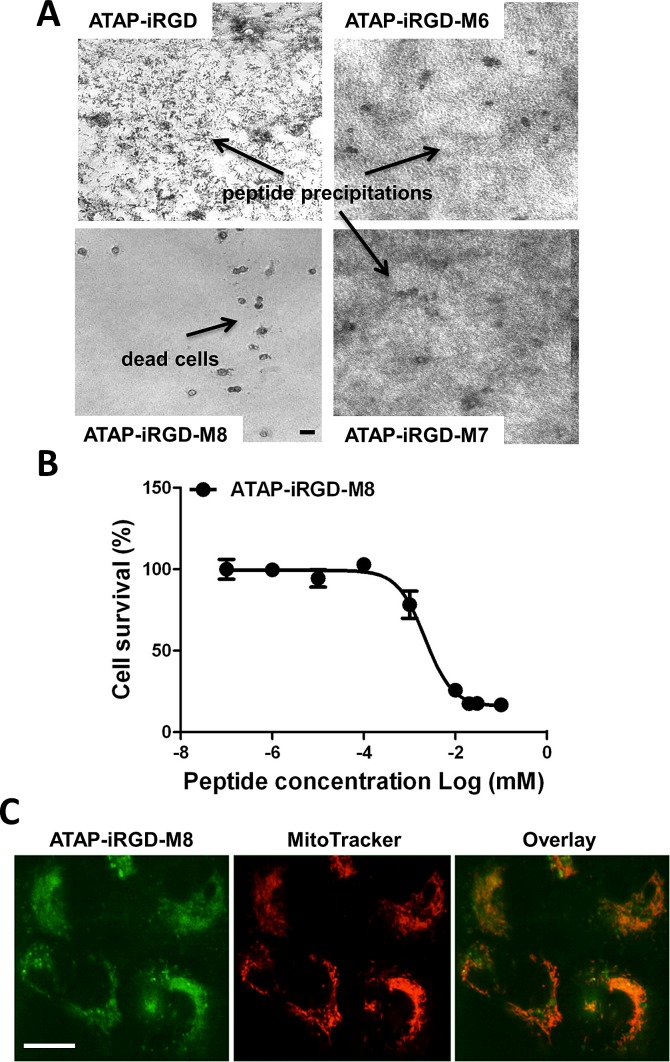
ATAP-iRGD-M8 displays more favorable property than ATAP-iRGD for treatment of prostate cancer cells (A) 200 μM indicated peptides were added to DU145 cells grown in a normal culture medium at 37 °C. ATAP-iRGD, ATAP-iRGD-M6 and M7 peptides all precipitate during 24 hours incubation, whereas the ATAP-iRGD-M8 shows no precipitation. The scale bar represents 20 μm. (B) Establishment of IC_50_ of ATAP-iRGD and ATAP-iRGD-M8 in DU145 cells by MTT assay. (C) Typical mitochondria localization pattern of ATAP-iRGD-M8 following incubation in DU145 cells. When applied externally to cells the Dylight488-labeled ATAP-iRGD-M8 (*Green*, *left*) can enter into cells and co-localized with mitochondria labeled with Mito Tracker Deep Red 633 (*Red, center*) as shown by the overlay image (*right*). The scale bar represents 20 μm.

Before advancing to *in vivo* tests, we need to solve some issues with the biophysical properties of ATAP-iRGD. The ATAP-iRGD peptide contains five acidic residues (three Glu and two Asp) and six basic residues (five Lys and one Arg) plus a hydrophobic [TFLEVTGKI] domain (Fig. 1A). At pH 7, the predicted dissociated charge of the peptide is +1, which limits its aqueous solubility in the physiological pH range. The isoelectric point of ATAP-iRGD is 9.5, thus the peptide will be more soluble in acidic solution. We have noticed that the ATAP-iRGD peptide tends to form aggregates when applied into cell culture medium with pH 6.5 to 7.5, at concentrations above 50 μM (Fig. 3A). This may decrease the bioactivity for ATAP-iRGD for intravenous administration due to potential precipitation in the blood circulation at a pH range of 7.2–7.4.

Toward improving the peptide solubility in the physiological pH environment, several modifications were introduced to the ATAP-iRGD sequence (Table 2). We first conducted experiments to neutralize the overall positive charge on ATAP-iRGD. For this purpose, point mutations were introduced at the first Lys residue, K1A (Mutant1, M1) and K1L (M2). Based on MTT assay with DU145 cells, we determined that the K1A and K1L peptide had IC_50_ of 36 μM and 68 μM, respectively, which were significantly higher than the parental ATAP-iRGD (IC_50_ = 1.6 μM). From this experiment, we conclude that the first Lys residue is critical to the pro-apoptotic activity for ATAP. Next, we generated two additional variants by neutralizing the Lys residue at position four and twenty-six, K4L (M3) and K26L (M4). These two mutants also caused significant reduction in the pro-apoptotic activity based on MTT assay. Essentially, the K26L mutant caused complete abolishment of the cytotoxic activity for ATAP-iRGD that we were unable to determine the IC_50_ value. We also generated another construct by replacing the nonpolar Met9 in the α-helix region with an acidic polar Glu. The M9E (M5) construct again showed significantly reduced cytotoxic activity compared with the original ATAP-iRGD peptide (Table 2, M5). The result with the K26L and M9E mutants further demonstrated that the amphipathic characteristic of ATAP is essential for the pro-apoptotic activity. Overall, our initial efforts to improve ATAP-iRGD solubility through elimination of the overall charge proved to be unsuccessful.

**Table 2:** Properties of the modified ATAP-iRGD peptides The parental ATAP-iRGD contains the C19A mutation (with A underlined). All substitutions in amino acids were underlined. Ac – acetylation; amide – amidation. IC_50_ were values derived from MTT assay with DU145 cells.

	Sequence	IC_50_	Theoretical pI	Net charge	Estimated half life (hrs)
Parental	KFEPKSGWMTFLEVTGKIAEMLSLLKQYCRGDKGPDC	1.6 μM	9.45	1	1.3
M1	AFEPKSGWMTFLEVTGKIAEMLSLLKQYCRGDKGPDC	36 μM	7.1	0	4.4
M2	LFEPKSGWMTFLEVTGKIAEMLSLLKQYCRGDKGPDC	68 μM	7.1	0	5.5
M3	KFEPLSGWMTFLEVTGKIAEMLSLLKQYCRGDKGPDC	25 μM	7.1	0	1.3
M4	KFEPKSGWMTFLEVTGKIAEMLSLLLQYCRGDKGPDC	>200 μM	7.01	0	1.3
M5	KFEPKSGWETFLEVTGKIAEMLSLLKQYCRGDKGPDC	6 μM	7.13	0	1.3
M6	KKFEPKSGWMTFLEVTGKIAEMLSLLKQYCRGDKGPDC	3.1 μM	9.82	2	1.3
M7	Ac-KFEPKSGWMTFLEVTGKIAEMLSLLKQYCRGDKGPDC-amide	2.1 μM	9.69	1	stable
M8	Ac-KKFEPKSGWMTFLEVTGKIAEMLSLLKQYCRGDKGPDC-amide	2.1 μM	10.02	2	stable

Since mutations within the ATAP sequence were not possible, we turned our attention to modification outside the ATAP sequence. One factor we need to consider is the half-life time for the ATAP-iRGD peptide in blood circulation. According to Protparam software, the estimated half-life of ATAP-iRGD is ~1.3 hours. Acetylation of the amino terminus and amidation of the carboxyl terminus have been shown to increase peptide stability by reducing enzymatic degradation [30, 38]. When these manipulations were introduced to the ATAP-iRGD peptide (M7 in Table 2), we found that the M7 peptide did not show improved solubility in aqueous solution, even though this amino- and carboxyl-modification should increase the half-life due to protection from ubiquitin mediated enzymatic degradation [39].

We reasoned that adding additional positively charged residues outside the ATAP sequence might improve the overall electrophilic property and thus the aqueous solubility for ATAP-iRGD. We included one more Lys from the original Bfl-1 sequence in front of ATAP that led to the generation of M6 and M8, which contains an overall net charge of +2. As in M7, the amino- and carboxyl-termini of M8 were protected by acetylation and amidation. Because we have not touched the ATAP sequence, the experimentally determined IC_50_ for M6, M7 and M8 peptides were essentially the same as that of the original ATAP-iRGD (Table 2). M6 and M7 constructs did not show significant improvement in aqueous solubility at neutral pH, since pronounced precipitation was observed when these peptides were dissolved in normal cell culture medium during 24 hours incubation (Fig. 3A). Excitingly, the M8 peptide showed markedly increased aqueous solubility at neutral pH, as no precipitation was observed at concentration above 200 μM with prolonged incubation in the normal cell culture medium. A reasonable explanation is that in M6 the carboxyl group contributed to a strong-acid character, and the amine group contributed to a weak base character, and substitution by acetylation and amidation in M8 would result in the whole peptide be more alkaline and thus more dissolvable in aqueous solution at neutral pH.

Encouraged by our finding that ATAP-iRGD-M8 could maintain the pro-apoptotic activity and at the same time with improved stability and aqueous solubility, we next sought to determine whether this peptide still maintains cytotoxicity and mitochondria targeting property. As shown in Fig. 3B, ATAP-iRGD-M8 has similar cytotoxic effect as ATAP-iRGD with an IC_50_ of 2.1±0.5 μM, while the unconjugated ATAP failed to kill DU145 cells (Fig. 2A). As shown in Fig. 3C, the Dylight488-labelled ATAP-iRGD-M8 showed clear mitochondria target pattern that displayed significant overlapping pattern with the Mito Tracker Red. Together, our results showed that the ATAP-iRGD-M8 peptide has better solubility and stability in physiological pH environment as compared to the original ATAP-iRGD.

### Antitumor effect of ATAP-iRGD-M8 in xenograft model

**Figure 4:**
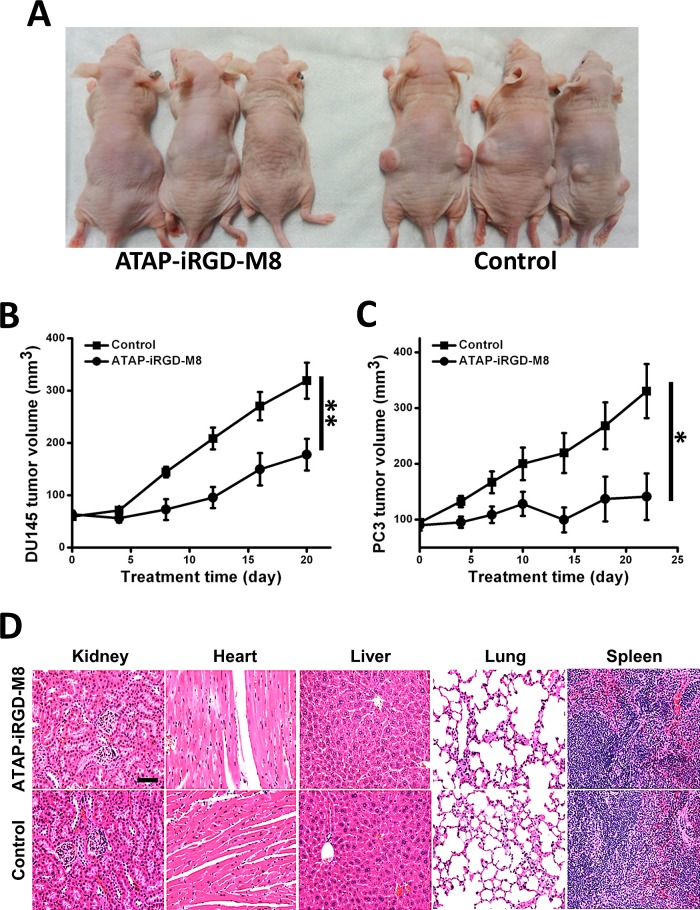
ATAP-iRGD-M8 suppresses prostate tumor growth with no toxic effects in xenograft model (A) DU145 cells were subcutaneously injected into both flanks of nude mice and allowed to establish xenografts for two weeks. 21 mg/kg of ATAP-iRGD-M8 or saline were administered into the mice via tail vein injection. Picture was taken at 16 days post treatment. (B) The DU145 xenograft volumes from animals treated with ATAP-iRGD-M8 were significantly smaller than those from animals treated with the control saline (ATAP-iRGD-M8 treatment group n=6, saline treatment group n=9). (C) Nude mice implemented with PC-3 cells also show significant suppression of tumor growth following treatment with ATAP-iRGD-M8. (D) Morphological details were investigated using H&E staining. No significant pathological changes in kidney, heart, liver, lung, and spleen were observed in nude mice following treatment with ATAP-iRGD. The scale bar represents 50 μm.

To test the *in vivo* antitumor efficacy of ATAP-iRGD-M8, we systemically delivered the peptide into nude mice implemented with either DU145 or PC-3 cells. The tumor sizes were measured during the different treatment times. Fig. 4A showed representative picture of nude mice implemented with the DU145 tumor at 16 days post treatment with ATAP-iRGD-M8 or saline as control. The time-dependent changes in tumor growth for DU145 and PC-3 in the nude mice were shown in Fig. 4B and 4C, respectively. Clearly, systemic delivery of ATAP-iRGD-M8 led to effective suppression of both DU145 and PC-3 tumor growth in the xenograft model. At the end of the xenograft experiment, xenograph from DU-145 cells were dissected and weighted. In Supplemental Fig. S2, we show that ATAP-iRGD-M8 significantly suppressed DU-145 tumor growth.

**Figure 5:**
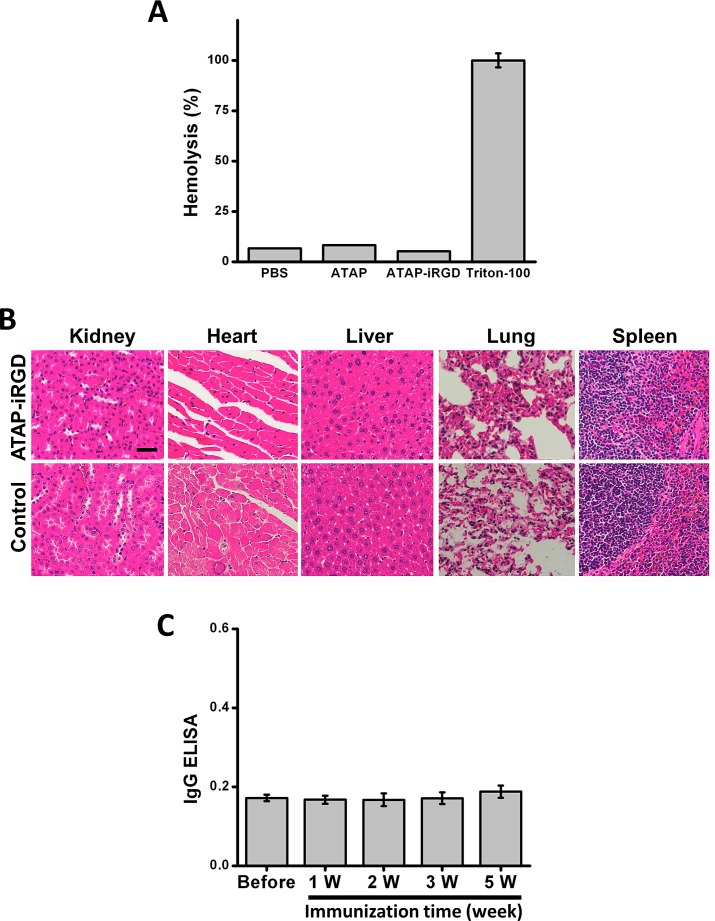
ATAP-iRGD has low immunogenicity in mouse model with minimum effect on hemolysis (A) Treatment of mouse red blood cells (RBCs) with 20 μM ATAP-iRGD did not cause hemolysis. PBS and 1%Triton-100 sample were used as negative and positive controls, respectively. Results were means of triplicates ±SEM. (B) Repetitive intravenous delivery of ATAP-iRGD did not produce obvious toxicity to vital organs in the SV129 mice. H&E staining of the ATAP-iRGD and control saline treatment mice show no significant pathological changes in kidney, heart, liver, lung and spleen. The scale bar represents 50 μm. (C) SV129 mice were immunized with 100 μg ATAP-iRGD-M8 daily for five weeks. Blood samples were collected 1, 2, 3, and 5 weeks after first immunization. Antibodies were detected on ATAP-iRGD-M8 coated plates, respectively, using ELISA against the mouse whole IgG. Error bars indicate ±SD (n=5).

To test the potential toxicity of ATAP-iRGD-M8 in the experimental mice, H&E-stained sections of the kidney, heart, liver, lung and spleen were examined after peptide treatment. As shown in Fig. 4D, there were no obvious changes in these organs following treatment with ATAP-iRGD-M8 as compared with control. We next used hemolysis assay to further investigate the potential toxicity of ATAP-iRGD using red-blood cells (RBC) derived from the wild type SV129 mice. Release of hemoglobin from erythrocytes following treatment with ATAP-iRGD was measured. For positive control, RBCs were treated with 1% Triton-100. Treatment with isotonic PBS is used as negative control. The data presented in Fig. 5A indicate that treatment with ATAP-iRGD did not cause hemolytic damage to RBCs, as the degree of peptide-induced hemolysis was nearly undetectable (similar to that caused by PBS). Thus, intravenous administration is a suitable choice of ATAP-iRGD delivery.

For further evaluation of the *in vivo* toxic effect of ATAP-iRGD, repetitive injections of ATAP-iRGD into the immune intact SV129 mice were conducted. The mice could tolerate ATAP-iRGD with normal growth pattern during the treatment period, with no visible signs or symptoms of toxicity in the treated animals. H&E staining revealed no significant pathological abnormalities in the kidney, heart, liver, lung and spleen (Fig. 5B).

In order to test whether the peptide has immunogenicity, the antibody level in serum obtained from the SV129 mice immunized with ATAP-iRGD-M8 was measured. As shown in Fig. 5C, antibody titers in sera from ATAP-iRGD-M8 treated mice were comparable with those sera collected before immunization. No obvious difference in mouse whole IgG production was observed before or after immunization over a 5-week experimental duration. This data indicates that ATAP-iRGD-M8 has low immunogenicity in mice.

## DISCUSSION

In this study, we conducted the proof-of-principle experiments for testing the therapeutic value for using ATAP-iRGD peptide as a cancer treatment agent. We showed that ATAP-iRGD is effective in suppressing growth of multiple cancer cell lines *in vitro*, including DU145 which has been proved to be resistant to BH3 derived peptide[36]. For improvement of solubility and bioactivity for ATAP-iRGD, we introduced several variants of ATAP-iRGD and arrived at a final ATAP-iRGD-M8 construct. The ATAP-iRGD-M8 peptide is soluble in physiological saline solution, and maintains potent pro-apoptotic activity in prostate cancer cells with limited off-target toxicity on normal tissues. We have worked out the protocol for scale-up production of the ATAP-iRGD-M8 peptide that can be used for further clinical studies.

Many chemical synthetic strategies have been used to improve peptide drug candidate because of low stability in plasma and sensitivity to proteases degradation, such as glycosylation, substituting D-amino acids for L-amino acids, and chemical blocking amino- or carboxy-terminal ends, etc. [2, 40]. In this study, we introduced acetylation at the amino-terminus and amidation at the carboxyl-terminus that leads to protection of the ATAP peptide from degradation by proteases, resulting in increase in half-life of the peptide in blood circulation. We also extended the original 29 amino acids in ATAP to include one additional Lys residue that is naturally present in Bfl1. Addition of this positive charge renders the ATAP-iRGD-M8 peptide to be more alkaline and electrophilic, thus leading to remarkable improvement in solubility of the peptide in physiological saline solution. We showed that ATAP-iRGD-M8 could target the tumor cell mitochondria with potent pro-apoptotic activity based on both *in vitro* and *in vivo* assays. iRGD-mediated targeting and delivery of ATAP into tumor cells allows for recognition of the cytotoxic agents by tumor tissues, reducing the off-target effects on non-tumor tissues. Our pathological evaluation and hemolysis assays show that ATAP-iRGD/ATAP-iRGD-M8 did not produce systemic side effect following repetitive intravenous deliveries.

A number of antitumor strategies target the Bcl-2 family protein, such as the use of BH3 peptide or mimetics, have been proposed for cancer treatment [30, 31]. The mechanism of action for BH3 peptides involves perturbation of the balance between the pro- and anti-apoptotic Bcl-2 family proteins in regulation of apoptosis of cancer cells [29]. Either down-regulation of pro-apoptotic or up-regulation of anti-apoptotic is common features of many cancer cell types, which can cause resistance to BH3 peptide-induced cell death. In this regard, the main advantage of ATAP over the BH3 peptide lies in the ability for ATAP to induce cell apoptosis independent of Bcl-2 family proteins [33, 35]. Our previous studies indicated that the pro-apoptotic function for ATAP was not affected in cells with overexpression of Bcl-2 or Bcl-xL or in cells lacking expression of Bax or Bak, whereas BH3 peptide was ineffective under these conditions [35]. Here we show that ATAP-iRGD-M8 effectively suppress Bax-deficient DU145 prostate cancer cells [36] in the xenograft model. Since impaired apoptosis by either overexpression of a pro-survival family member or loss of a pro-apoptotic relative is both commonly observed in cancer cells and often causes resistance to treatment, our data indicates that ATAP-iRGD-M8 has potential to overcome this major barrier for treatment of broader cancer types.

Another question related to development of peptide drugs in cancer treatment is the assessment of the potential immunogenic nature of the peptide sequence. Since systemic delivery of peptide-drug most likely involves repetitive administrations, therapeutic peptides containing potential immunogenic sequences is one of the major issues that preventing them from becoming successful drug candidates. Here we show that ATAP-iRGD-M8 had no obvious immunogenicity when tested during a five-week immunization in the rodent model. Such data support further development in clinical settings. Clearly, due to the differences between rodent and human, more critical assessment of the immunogenic property for ATAP-iRGD-M8 will be required in future clinical trials prior to further evaluation of the efficacy for ATAP-iRGD in human studies.

ATAP was originally identified as a conserved pro-apoptotic function domain of the Bfl-1 protein [34]. The ATAP sequence contains a unique tail-anchoring motif that enables targeting to the mitochondria outer membrane. This property provides another safety feature for the use of ATAP-iRGD-M8 peptide in cancer treatment, since the peptide does not appear to interfere with the permeability of the plasma membrane. Thus, prior to delivery into the tumor cells, ATAP-iRGD-M8 should not produce cytotoxic effect on normal tissues. Specific targeting of ATAP to mitochondria would likely require association with other membrane-anchoring proteins, rather than direct association with membrane lipid. In future studies, it will be important to determine the mechanisms that underlie the functional interaction ATAP and its associated proteins in regulating the mitochondria permeability transition associated with cell growth and death.

## MATERIALS AND METHODS

### Peptide design and synthesis

A synthetic ATAP peptide, KFEPKSGWMTFLEVTGKIAEMLSLLKQYC, was coupled to the tumor-homing peptide iRGD [CRGDKGPDC], by omitting one overlapping cysteine. The amino acid compositions for ATAP-iRGD and related variations are listed in Table 2. For comparative studies, unconjugated ATAP, BH3-iRGD [MGQVGRQLAIIGDDINRRYDSCRGDKGPDC] containing the human Bak-BH3 domain and iRGD was also synthesized. Peptides were synthesized at GL Biochem (GL Biochem Ltd, Shanghai, China) using Fmoc solid-phase synthesis method and purified by high-performance liquid chromatography (HPLC) using H_2_O-trifluoroacetic acid/acetonitrile gradient, and confirmed by ion-spray mass spectrometry. Greater than 95% purity was achieved and determined by HPLC. Characterizations of the physical and chemical parameters of these peptides, such as amino acid composition, molecular weight, isoelectric point (pI), half-life estimation and overall charge of peptide, were analyzed by the ExPASy's ProtParam tool (http://us.expasy.org/tools/protparam.html) and Peptide Property Calculator (http://pepcalc.com/protein-calculator.php).

### Cell culture and viability assay

The human esophageal squamous carcinoma cell line KYSE-150 was provided by Dr. Chung S. Yang (Rutgers University, Piscataway, NJ)[41, 42]. DU145, LNCaP, PC-3, K562, U87 and MDA-MB-231 cells were purchased from American Type Culture Collection and cultured with designated medium, supplemented with 10% fetal bovine serum, 100 unit/ml penicillin, and 100 μg/ml streptomycin. Cell viability was determined by MTT assay. Cells were plated at 1,000-1,500 cells per well in a 96-well plate and then treated with indicated amounts of ATAP peptides for 48 hours. Then, 20 μl (5 mg/ml in PBS) MTT [3-(4,5-dimethylthiazol-2-yl)-2,5-diphenyltetrazolium bromide] (Sigma Chemical Co., St. Louis, Mo.) was added to each well. After another 4 hours, 150 μL DMSO was added to dissolve formazan crystals in each well, and the absorbance were recorded at 570 nm.

### Cytochrome c release from mitochondria

Mitochondria were isolated from cells following the protocol of Unkila et al.[43]. DU145 cells (1×10^7^) were incubated in medium with or without 20 μM ATAP-iRGD. After 6 hours, cells were washed three times with ice-cold phosphate-buffered saline (PBS) and suspended in a solution containing 250 mM sucrose, 20 mM HEPES, 1 mM dithiothreitol, 10 mM KCl, 1 mM EDTA, 1 mM EGTA, 1.5 mM MgCl_2_, and a mixture of protease inhibitors at 4 °C. Cells were then homogenized with 20 strokes in a 22-gauge syringe and centrifuged at 800*g* for 10 min to remove intact cells and nuclei. Microsomal membranes containing mitochondria were pelleted by centrifugation at 15,000*g* for 10 min at 4 °C, and resuspended in RIPA buffer (150 mM NaCl, 5 mM EDTA, 1% Nonidet P-40, 20 mM Tris-HCl, pH7.5). For each sample, 40 μg of total protein from the cytosolic (supernatant) and mitochondrial fraction (pellet) were applied to 15% SDS-PAGE and probed with anti-cytochrome c antibody (BD Biosciences, San Jose, CA), with anti-β-actin as loading controls.

### Live-cell imaging

ATAP-iRGD and ATAP-iRGD-M8 peptides were labeled with an amine-reactive DyLight488 fluorophore (Pierce Biotechnology, Rockford, USA) according to the manufacturer's instructions. DU145 cells were cultured in the presence of 2 μM Dylight488-labeled ATAP-iRGD or ATAP-iRGD-M8 plus 50 nM Mito Tracker Red (Molecular Probes Inc., Eugene, OR). 50 μM Q-VD-OPh was added to the culture medium in order to prevent cell apoptosis. Sample images were taken by BioRad 2100 Radiance laser scanning confocal microscope (Bio-Rad Laboratories, Hercules, CA) with a 60×, 1.3NA oil immersion objective (Nikon Instruments Inc., Melville, NY).

### Soft agar assay for tumor colony growth

The effect of ATAP-iRGD peptide on tumor cell growth was measured by soft agar assay. The bottom agar layer was prepared in 12 well plates by adding 0.8 ml of 1.4% agar (Difco Laboratories, Detroit, MI) mixed with 0.8 ml 2X cell growth medium with required amount of peptides. Then, 0.8 ml of 1% agar mixed with 0.8ml 2X cell growth medium containing 2000 DU145 cells and required amount of peptide were added on top of the solidified agar layer so that the final peptide concentration on both layers is 10 μM. Cells were fed for 2-3 times per week with cell culture medium with 10 μM peptide. After 14 days, cells were stained with 1% crystal violet and colony numbers were quantitated using Quantity One software (Bio-Rad, Hercules, CA).

### Xenograft studies in nude mice

Handling of animals was performed in accordance with protocols approved by the Institutional Animal Care and Use Committee (IACUC) of the Ohio State University. Five week-old NCR nude mice (Taconic Farms, Germantown, NY) were implanted subcutaneously in both flanks with 2 ×10^6^ DU145 or PC-3 cells. After tumors reached 4 to 7 mm in diameter (about 9~14 days after implantation), the mice were randomly divided into two groups so that both the mean and the variance of the tumor diameters are of no significant difference among the groups prior to treatment. The ATAP-iRGD-M8 peptide was injected through tail vein once every two days during the whole procedure. Tumor volume was measured by a digital caliper (Thermo Fisher Scientific, Waltham, MA). Tumor volume was determined from the orthogonal dimensions (*length, width*) using the formula: *tumor volume* = 1/2(*length* × *width*^2^) [42, 44]. According to the IACUC guideline, the experiments will be terminated when tumors reached 1.5 cm in diameter. At the end of experiment, mice were sacrificed and xenografts were removed and weighted. For toxicological evaluation of peptide treatments, mouse body weights were measured every four days. In addition, after euthanizing animals, organs (kidney, heart, liver, lung and spleen) were expanded, fixed in 10% neutral buffered formalin, paraffin embedded, and stained with hematoxylin-eosin (H&E) for pathological analysis.

### Red blood cell (RBC) hemolysis assay

RBCs from SV129 mice were harvested by centrifugation of the whole blood for 5 min at 3000 rpm, washed three times with 150 mM NaCl, and resuspended in the pH7.4 100 mM phosphate buffer (PB, isotonic solution). RBC was diluted with same buffer to yield a suspension of ~5×10^8^ cells/ml. 1×10^8^ RBCs were incubated with or without 20 μM peptide present in the PB buffer at 37 °C for 1 hour. RBCs were then spun down, and lysis of the cells was determined by measuring the absorbance of the supernatant at 541 nm for quantification of the amount of hemoglobin that appeared in the supernatant. Hemolysis percentage was calculated by assuming 100% hemolysis to be measured by the hemoglobin released by the red blood cells in solution containing 1% Triton-X100; negative control of RBCs in buffer with PBS was also tested.

Calculation of percentage of hemolysis:

Hemolysis (%) = (*Absorbance_sample_*) - (*Absorbance_blank_*) / (*Highest Absorbance_positive control(Water)_ Triton-X100) ×*100

### Immunogenicity assay of ATAP-iRGD in mice

Male SV129 mice were immunized subcutaneously on day 0, 7, 14 and 21 with 100 μg ATAP-iRGD-M8 peptide. Blood was withdrawn for analysis before and once per week for 5 weeks after immunization. Whole IgG levels from the mice serum were assessed by a solid-phase ELISA on microtiter plates (Nunc, Roskilde, Denmark).

### Statistical analysis

Statistical analyses were performed using GraphPad Prism version 4.0 (GraphPad Software, San Diego, CA, USA). Data are presented as means ± SD/SEM. Statistical analysis was performed using two-tailed Student's t test, and p value <0.05 was considered of significant difference.

## SUPPLEMENTARY MATERIAL FIGURES



## Abbreviations

ATAP: amphipathic tail-anchoring peptide
PBS: phosphate-buffered saline
BH3: Bcl-2 homology domain-3

## References

[1] Lien S, Lowman HB (2003). Therapeutic peptides. Trends in biotechnology.

[2] Vlieghe P, Lisowski V, Martinez J, Khrestchatisky M (2010). Synthetic therapeutic peptides: science and market. Drug discovery today.

[3] Thundimadathil J (2012). Cancer treatment using peptides: current therapies and future prospects. Journal of amino acids.

[4] Mulder KC, Lima LA, Miranda VJ, Dias SC, Franco OL (2013). Current scenario of peptide-based drugs: the key roles of cationic antitumor and antiviral peptides. Frontiers in microbiology.

[5] Gurpinar E, Grizzle WE, Piazza GA (2014). NSAIDs inhibit tumorigenesis, but how?. Clinical cancer research : an official journal of the American Association for Cancer Research.

[6] Jacotot E, Deniaud A, Borgne-Sanchez A, Touat Z, Briand JP, Le Bras M, Brenner C (2006). Therapeutic peptides: Targeting the mitochondrion to modulate apoptosis. Biochimica et biophysica acta.

[7] Curnis F, Sacchi A, Borgna L, Magni F, Gasparri A, Corti A (2000). Enhancement of tumor necrosis factor alpha antitumor immunotherapeutic properties by targeted delivery to aminopeptidase N (CD13). Nature biotechnology.

[8] Arap W, Pasqualini R, Ruoslahti E (1998). Cancer treatment by targeted drug delivery to tumor vasculature in a mouse model. Science.

[9] Koivunen E, Arap W, Valtanen H, Rainisalo A, Medina OP, Heikkila P, Kantor C, Gahmberg CG, Salo T, Konttinen YT, Sorsa T, Ruoslahti E, Pasqualini R (1999). Tumor targeting with a selective gelatinase inhibitor. Nature biotechnology.

[10] Pasqualini R, Ruoslahti E (1996). Organ targeting in vivo using phage display peptide libraries. Nature.

[11] Ruoslahti E (1996). RGD and other recognition sequences for integrins. Annual review of cell and developmental biology.

[12] Pytela R, Pierschbacher MD, Ruoslahti E (1985). Identification and isolation of a 140 kd cell surface glycoprotein with properties expected of a fibronectin receptor. Cell.

[13] Koivunen E, Gay DA, Ruoslahti E (1993). Selection of peptides binding to the alpha 5 beta 1 integrin from phage display library. The Journal of biological chemistry.

[14] Healy JM, Murayama O, Maeda T, Yoshino K, Sekiguchi K, Kikuchi M (1995). Peptide ligands for integrin alpha v beta 3 selected from random phage display libraries. Biochemistry.

[15] Danhier F, Le Breton A, Preat V (2012). RGD-based strategies to target alpha(v) beta(3) integrin in cancer therapy and diagnosis. Molecular pharmaceutics.

[16] Enback J, Laakkonen P (2007). Tumour-homing peptides: tools for targeting, imaging and destruction. Biochemical Society transactions.

[17] Ye Y, Zhu L, Ma Y, Niu G, Chen X (2011). Synthesis and evaluation of new iRGD peptide analogs for tumor optical imaging. Bioorganic & medicinal chemistry letters.

[18] Sugahara KN, Teesalu T, Karmali PP, Kotamraju VR, Agemy L, Girard OM, Hanahan D, Mattrey RF, Ruoslahti E (2009). Tissue-penetrating delivery of compounds and nanoparticles into tumors. Cancer Cell.

[19] Teesalu T, Sugahara KN, Kotamraju VR, Ruoslahti E (2009). C-end rule peptides mediate neuropilin-1-dependent cell, vascular, and tissue penetration. Proceedings of the National Academy of Sciences of the United States of America.

[20] Sugahara KN, Teesalu T, Karmali PP, Kotamraju VR, Agemy L, Greenwald DR, Ruoslahti E (2010). Coadministration of a tumor-penetrating peptide enhances the efficacy of cancer drugs. Science.

[21] Constance JE, Lim CS (2012). Targeting malignant mitochondria with therapeutic peptides. Therapeutic delivery.

[22] Fulda S, Galluzzi L, Kroemer G (2010). Targeting mitochondria for cancer therapy. Nature reviews Drug discovery.

[23] Hockenbery DM (2010). Targeting mitochondria for cancer therapy. Environmental and molecular mutagenesis.

[24] Javadpour MM, Juban MM, Lo WC, Bishop SM, Alberty JB, Cowell SM, Becker CL, McLaughlin ML (1996). De novo antimicrobial peptides with low mammalian cell toxicity. Journal of medicinal chemistry.

[25] Ellerby HM, Arap W, Ellerby LM, Kain R, Andrusiak R, Rio GD, Krajewski S, Lombardo CR, Rao R, Ruoslahti E, Bredesen DE, Pasqualini R (1999). Anti-cancer activity of targeted pro-apoptotic peptides. Nature medicine.

[26] Smolarczyk R, Cichon T, Graja K, Hucz J, Sochanik A, Szala S (2006). Antitumor effect of RGD-4C-GG-D(KLAKLAK)2 peptide in mouse B16(F10) melanoma model. Acta biochimica Polonica.

[27] Walensky LD, Kung AL, Escher I, Malia TJ, Barbuto S, Wright RD, Wagner G, Verdine GL, Korsmeyer SJ (2004). Activation of apoptosis in vivo by a hydrocarbon-stapled BH3 helix. Science.

[28] Willis SN, Fletcher JI, Kaufmann T, van Delft MF, Chen L, Czabotar PE, Ierino H, Lee EF, Fairlie WD, Bouillet P, Strasser A, Kluck RM, Adams JM, Huang DC (2007). Apoptosis initiated when BH3 ligands engage multiple Bcl-2 homologs, not Bax or Bak. Science.

[29] Letai A, Bassik MC, Walensky LD, Sorcinelli MD, Weiler S, Korsmeyer SJ (2002). Distinct BH3 domains either sensitize or activate mitochondrial apoptosis, serving as prototype cancer therapeutics. Cancer cell.

[30] Walensky LD, Pitter K, Morash J, Oh KJ, Barbuto S, Fisher J, Smith E, Verdine GL, Korsmeyer SJ (2006). A stapled BID BH3 helix directly binds and activates BAX. Molecular cell.

[31] Oltersdorf T, Elmore SW, Shoemaker AR, Armstrong RC, Augeri DJ, Belli BA, Bruncko M, Deckwerth TL, Dinges J, Hajduk PJ, Joseph MK, Kitada S, Korsmeyer SJ, Kunzer AR, Letai A, Li C (2005). An inhibitor of Bcl-2 family proteins induces regression of solid tumours. Nature.

[32] van Delft MF, Wei AH, Mason KD, Vandenberg CJ, Chen L, Czabotar PE, Willis SN, Scott CL, Day CL, Cory S, Adams JM, Roberts AW, Huang DC (2006). The BH3 mimetic ABT-737 targets selective Bcl-2 proteins and efficiently induces apoptosis via Bak/Bax if Mcl-1 is neutralized. Cancer cell.

[33] Ko JK, Choi KH, Pan Z, Lin P, Weisleder N, Kim CW, Ma J (2007). The tail-anchoring domain of Bfl1 and HCCS1 targets mitochondrial membrane permeability to induce apoptosis. Journal of cell science.

[34] Ko JK, Choi KH, Kim HJ, Choi HY, Yeo DJ, Park SO, Yang WS, Kim YN, Kim CW (2003). Conversion of Bfl-1, an anti-apoptotic Bcl-2 family protein, to a potent pro-apoptotic protein by fusion with green fluorescent protein (GFP). FEBS letters.

[35] Ko JK, Choi KH, Peng J, He F, Zhang Z, Weisleder N, Lin J, Ma J (2011). Amphipathic tail-anchoring peptide and Bcl-2 homology domain-3 (BH3) peptides from Bcl-2 family proteins induce apoptosis through different mechanisms. The Journal of biological chemistry.

[36] Song JH, Kandasamy K, Kraft AS (2008). ABT-737 induces expression of the death receptor 5 and sensitizes human cancer cells to TRAIL-induced apoptosis. The Journal of biological chemistry.

[37] Guerrero CA, Mendez E, Zarate S, Isa P, Lopez S, Arias CF (2000). Integrin alpha(v)beta(3) mediates rotavirus cell entry. Proceedings of the National Academy of Sciences of the United States of America.

[38] Yang JZ, Chen W, Borchardt RT (2002). In vitro stability and in vivo pharmacokinetic studies of a model opioid peptide, H-Tyr-D-Ala-Gly-Phe-D-Leu-OH (DADLE), and its cyclic prodrugs. The Journal of pharmacology and experimental therapeutics.

[39] Hershko A, Heller H, Eytan E, Kaklij G, Rose IA (1984). Role of the alpha-amino group of protein in ubiquitin-mediated protein breakdown. Proceedings of the National Academy of Sciences of the United States of America.

[40] Antosova Z, Mackova M, Kral V, Macek T (2009). Therapeutic application of peptides and proteins: parenteral forever?. Trends in biotechnology.

[41] Hou Z, Sang S, You H, Lee MJ, Hong J, Chin KV, Yang CS (2005). Mechanism of action of (-)-epigallocatechin-3-gallate: auto-oxidation-dependent inactivation of epidermal growth factor receptor and direct effects on growth inhibition in human esophageal cancer KYSE 150 cells. Cancer research.

[42] Zhu H, Zhang H, Jin F, Fang M, Huang M, Yang CS, Chen T, Fu L, Pan Z (2014). Elevated Orai1 expression mediates tumor-promoting intracellular Ca2+ oscillations in human esophageal squamous cell carcinoma. Oncotarget.

[43] Unkila M, McColl KS, Thomenius MJ, Heiskanen K, Distelhorst CW (2001). Unreliability of the cytochrome c-enhanced green fluorescent fusion protein as a marker of cytochrome c release in cells that overexpress Bcl-2. The Journal of biological chemistry.

[44] Feldman JP (2009). A mathematical model for tumor volume evaluation using two-dimensions. J Appl Quan Meth.

