# Supplementary material for: Amphipathic tail-anchoring peptide is a promising therapeutic agent for prostate cancer treatment
Source: Oncotarget. 2014 Jul 31;5(17):7734–47. doi: 10.18632/oncotarget.2301 (PMC4202157; doi:10.18632/oncotarget.2301)
Supplement: Supplementary file 1 [file oncotarget-05-7734-s001.pdf]

# Amphipathic tail-anchoring peptide is a promising therapeutic agent for prostate cancer treatment

## Supplementary Material

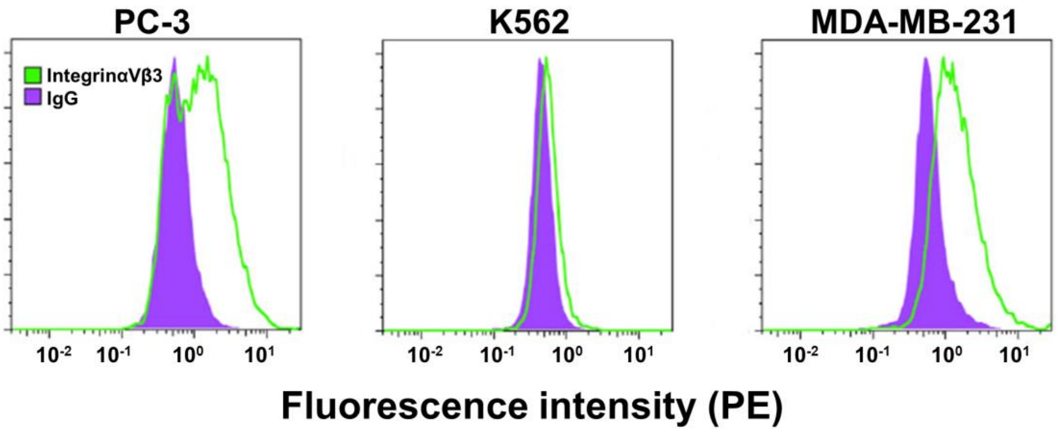


**Supplementary Figure S1: Flow cytometry analysis for expression of integrin aV(33 receptor in different cancer cell lines.**K562, PC-3 and MDA-MB-231 were subjected to flow cytometer analysis of expression of integrin aV 3- Cells were harvested and washed with PBS, resuspended in PBS and fixed with 4% formaldehyde. Cells were then stained with the following antibodies: Phycoerythrin (PE)-anti-integrin aV3 antibody (R&D Systems, Minneapolis, MN) or lgG isotype control. After washing twice with PBS, cells were analyzed by flow cytometer. Fluorescence of PE represents intensity of integrin aV 3- lgG signal was used as a negative control. The result showed that K562 expresses low level of integrin aV 3, and are resistant to ATAP-iRGD induced apoptosis (see **Table 1**).

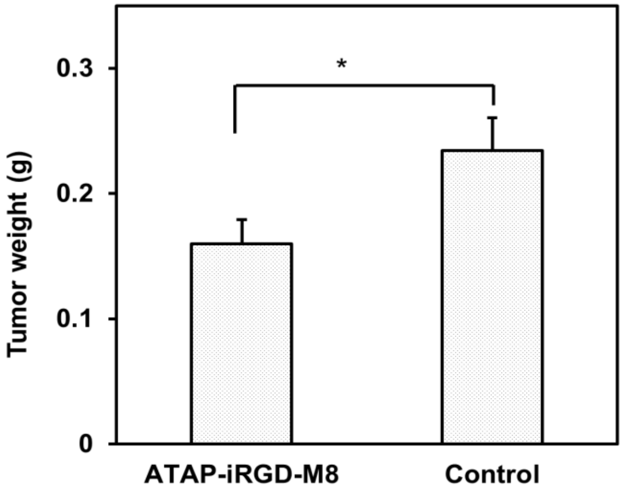


**Supplementary Figure S2: ATAP-iRGD-MB inhibits growth of DU-145 xenograft.**At the end of xenograft experiments, xenograft from DU-145 cells were dissected and weighted. ATAP-iRGD-M8 significantly suppressed xenograft growth. Results were mean±SEM. *: $P<0.05$.
